# Development of pXeDNA3.1-LacZ, a Dual-System Plasmid for Membrane Protein Expression in *Xenopus* and Mammalian Cells

**DOI:** 10.17912/micropub.biology.001866

**Published:** 2025-12-01

**Authors:** Li Guan, Tom E. Reynoldson, Eleonora M. Pieroni, James C. Dillon, Iris Hardege, Luis A. Yanez-Guerra

**Affiliations:** 1 School of biological sciences, University of Southampton, Southampton, England, United Kingdom; 2 Department of Zoology, University of Cambridge, Cambridge, England, United Kingdom

## Abstract

Cross-species expression of proteins in mammalian cells and *Xenopus* oocytes remains a powerful strategy for functional studies. This can be facilitated by plasmids with dual promoters, enabling use in both systems without separate subcloning. We developed pXeDNA3.1-LacZ, a plasmid that supports expression in mammalian cells and oocytes. The construct combines a cytomegalovirus promoter for mammalian transcription with *Xenopus* β-globin untranslated regions to enhance oocyte translation, and incorporates XcmI-mediated TA cloning and blue/white selection. To validate it, we used human TRPV1 (HsTRPV1). The vector drove robust expression in HEK293G5A cells and *Xenopus* oocytes.pXeDNA3.1-LacZ streamlines assays and facilitates studies of membrane proteins.

**Figure 1. Development and functional validation of the pXeDNA3.1-LacZ plasmid:**
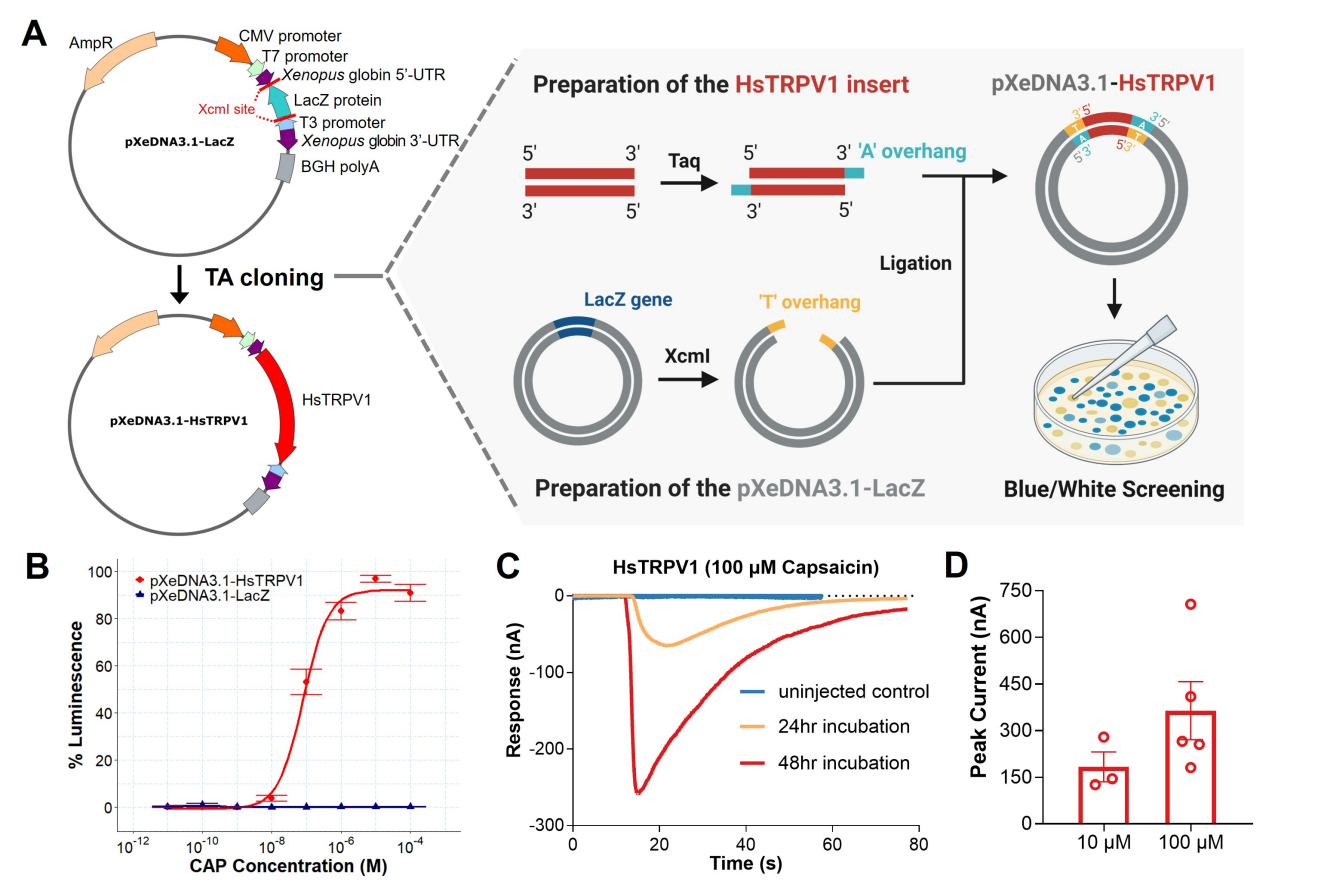
**A.** Schematic representation illustrating how the TA cloning strategy can be used to insert gene of interest (HsTRPV1) into the dual-expression vector pXeDNA3.1-LacZ. The plasmid was linearized by digestion with XcmI to generate T-overhangs, while the HsTRPV1 insert was amplified from U373-MG cell-derived cDNA and subsequently incubated with Taq DNA polymerase to generate complementary A-overhangs. Ligation products were mixed and following ligation the mix transformed into pXeDNA3.1-LacZ and plated onto selective media for blue/white screening. **B** . Dose-response curve obtained from bioluminescence assays in HEK293G5A cells transiently transfected with pXeDNA3.1-HsTRPV1, demonstrating dose dependent receptor activation by capsaicin (CAP). The half-maximal effective concentration (EC50) for capsaicin was determined as 0.103 μM. Data points represent mean ± SEM from three independent transfections, each performed in technical triplicates (n = 9 per concentration). **C** . Representative electrophysiological recordings obtained by two-electrode voltage clamp (TEVC) in *Xenopus* oocytes expressing HsTRPV1 24h and 48h after injection, showing characteristic inward currents upon application of 100 μM capsaicin (CAP). **D.** Peak currents from oocytes expressing HsTRPV1, 48 h post-injection, evoked by 10 μM and 100 μM capsaicin. Error bars represent the S.E.M. of three and five replicates, respectively.

## Description

The study of membrane proteins, particularly ion channels and G-protein coupled receptors (GPCRs), requires systems that allow integrated investigation of their structural, functional, and pharmacological properties. These proteins are central to cellular signalling, homeostasis, and disease mechanisms (Hilton et al., 2015; Caterina and Pang, 2016; Balse and Boycott, 2017; Sheng et al., 2017). *Xenopus* oocytes have served as a gold standard for functional expression, offering exceptional utility in electrophysiology, robustness in protein translation, and suitability for high-throughput screening of ion channels and ligand-gated receptors (Dascal, 1987; Stühmer, 1998; Hardege et al., 2023). By contrast, mammalian cell systems, such as HEK293 cells, provide a more physiologically relevant environment for investigating vertebrate signalling pathways, post-translational modifications, and subcellular trafficking (Thomas and Smart, 2005). Relying on a single system can be misleading because of species-specific contexts. For ion channels, mammalian cells better capture human-relevant trafficking and accessory subunit interactions. For example, in *Xenopus* oocytes, endogenous KCNE/MinK-related peptides (MiRPs) can modulate mammalian K⁺ channels, and trafficking-defective hERG mutants may appear functional in oocytes yet are retained in mammalian cells (Ficker et al., 2000; Anantharam et al., 2003; Lin et al., 2010). For GPCRs, G protein-coupled receptor kinase (GRK)/β-arrestin-dependent desensitisation and internalisation are most faithfully studied in mammalian cells, whereas oocytes often require co-expression of GRKs and β-arrestins to reconstitute these pathways (Kovoor et al., 1997, 1998; Celver et al., 2001). However, mammalian systems also have caveats. HEK293 cells exhibit an endogenous proton-gated conductance attributable to hASIC1a, which can confound studies of proton-gated receptors (Gunthorpe et al., 2001). They also express endogenous voltage-gated K⁺ channels (Yu and Kerchner, 1998). These channels recover from closed-state inactivation during the hyperpolarising prepulses used to activate hyperpolarisation-activated cyclic nucleotide-gated (HCN) channels; the subsequent positive tail step evokes outward K⁺ currents that can be misattributed to HCN channels, leading to inaccurate estimates of exogenous activity (Varghese et al., 2006). Together, these examples argue for complementary use of *Xenopus* oocytes and mammalian cells.

Transitioning between these models can be hindered by the need to subclone into separate vectors. Dual-expression plasmids overcome this by enabling side-by-side use of the same construct in *Xenopus* oocytes and mammalian cells. The pXOOM vector established this concept (Jespersen et al., 2002). Unfortunately there is no publicly accessible repository entry for an empty pXOOM backbone, nor a complete, downloadable plasmid map and sequence. Existing Addgene records contain only insert-bearing derivatives of pXOOM with limited documentation.

To mitigate this accessibility gap, we developed a dual-promoter plasmid for seamless expression in mammalian cells and *Xenopus* oocytes. The vector pairs a CMV promoter for robust mammalian transcription with *Xenopus* β-globin 5′/3′ UTRs to enhance oocyte translation and mRNA stability (Jespersen et al., 2002). An adjacent T7 promoter positioned immediately upstream of the β-globin 5′ UTR enables *in vitro* transcription of RNA for oocyte injection. For *in vitro* transcription, the plasmid can be linearised downstream of the β-globin 3′ UTR, using unique restriction sites (AgeI, ApaI, or PmeI). This ensures that capped transcripts include the complete 3′ UTR and its encoded 17-adenosine polyA tail. The bovine growth hormone polyadenylation signal, located further downstream of the *Xenopus* regions, functions specifically in mammalian cells to ensure proper mRNA processing. Whole-plasmid sequencing confirmed the final construct, designated pXeDNA3.1-LacZ (Figure 1). The plasmid and whole sequence are available in Addgene (ID; 242746).

To validate cross-system performance, we cloned HsTRPV1 into pXeDNA3.1-LacZ ( Figure 1A ) and assayed function in the two systems. In the mammalian cell line HEK293, HsTRPV1 activity was monitored using an aequorin-based calcium reporter, in which Ca²⁺ binding to the photoprotein triggers oxidation of coelenterazine and emission of bioluminescence. The stable aequorin-expressing HEK293 cell line used for these assays is referred to as HEK293G5A (Baubet et al., 2000; Thiel, Yañez-Guerra and Jekely, 2023). Since HsTRPV1 is a non-selective cation channel permeable to Ca²⁺, capsaicin application produces Ca²⁺ influx that is captured by calcium reporters, as shown before (Starkus et al *.* , 2019). Capsaicin was tested over a concentration range of 10⁻⁴ to 10⁻¹¹ M, producing graded bioluminescent signals corresponding to Ca²⁺ influx, from which a concentration–response curve with an EC₅₀ of 0.103 μM was derived ( Figure 1B ). This value falls within the range of those previously reported for HsTRPV1 expressed in HEK293 cells (pEC₅₀ ≈ 6.8; EC₅₀ ≈ 0.158 μM; Lam, McDonald and Lambert, 2005) and comparable to values obtained in CHO cells where HsTRPV1 was expressed using a mammalian-only expression vector (EC₅₀ = 0.53 μM; McIntyre et al., 2001). The addition of capsaicin in the empty-vector transfected HEK293G5A cells produced no signal ( Figure 1B ). To assess function in *Xenopus laevis* oocytes, we used two-electrode voltage clamp (TEVC). The addition of capsaicin (100 µM) to uninjected oocytes produced no detectable response ( Figure 1B ). However, robust inward currents were observed upon application of 10 µM and 100 µM capsaicin, 24 and 48 hours post-injection ( Figure 1C –D). At 48 hours, the mean peak current amplitudes were 0.18 ± 0.05 µA (10 µM) and 0.36 ± 0.09 µA (100 µM; values represent mean ± SEM). These values fall within the 0.1–15 µA range previously reported for rat TRPV1 expressed in oocytes and activated with 15 µM capsaicin (Rivera-Acevedo et al *.* , 2013). Because no equivalent quantitative data exist for human TRPV1 in oocytes, we used the Rivera-Acevedo et al *.,* dataset as a comparison, as it defines the expected range of TRPV1 current amplitudes across expression levels under systematically varied mRNA concentrations and incubation times. Our amplitudes are near the lower threshold of this range, corresponding to moderate expression levels, as reported by Rivera-Acevedo et al., for rat TRPV1, but remain well within the expected variability.

Our plasmid enables direct expression of identical constructs in both *Xenopus* oocytes and mammalian cells, avoiding additional subcloning and allowing functional differences to be attributed to the biological systems rather than to vector design. It also includes practical features such as TA cloning and blue/white screening, and we provide the full sequence and annotated map. Both the empty backbone and validation plasmid are freely available through Addgene (ID 242746), ensuring transparency and broad accessibility for future use.

A potential limitation of this study is that we tested the plasmid with only one receptor; HsTRPV1. However, our plasmid contains all the necessary regulatory elements for expression in both systems, as described above: a CMV promoter and bovine growth hormone (BGH) polyadenylation signal for mammalian expression, and *Xenopus* β-globin 5′ and 3′ untranslated regions (UTRs) for oocyte expression, followed by a 17-adenosine poly(A) stretch downstream of the 3′ UTR. This 17-adenosine tail lies above the minimal length required for high-affinity binding of cytoplasmic poly(A)-binding protein (PABPC), which typically needs a minimum of ~12 adenosines for stable interaction through RNA-recognition motifs 1 and 2 facilitating the translation (Kühn & Wahle, 2004; Mangus et al., 2003). However, in *Xenopus* oocytes, extending poly(A) tails has been shown to further enhance mRNA stability and translation; for example, artificial elongation of the *c-mos* tail directly activated its translation and meiotic maturation (Barkoff et al., 1998). Moreover, extending poly(A) tails on synthetic RNAs enhances their persistence after injection into *Xenopus* : capped, polyadenylated transcripts show markedly greater stability, with long (~200 nt) tails roughly doubling mRNA half-life compared with short-tailed or non-adenylated RNAs (Drummond et al., 1985; Harland & Misher, 1988; Colman & Drummond, 1986). While in vitro–transcribed RNA generated directly from our plasmid was sufficient to support robust TRPV1 expression in *Xenopus* , users who observe low expression in their own constructs should consider post-polyadenylating the transcripts using bacterial poly(A) polymerase (e.g. NEB M0276 or Thermo Fisher Poly(A) Tailing Kit), as extending the poly(A) tail can enhance mRNA stability and translation efficiency.

**Data availability.** Annotated SnapGene map; and analysis code are deposited at Addgene (pXeDNA3.1-LacZ, **#242746** ; pXeDNA3.1-HsTRPV1, **#244454** ). Raw data for the traces is available upon request.

**Ethics.** Defolliculated *Xenopus laevis* oocytes were purchased from EcoCyte Bioscience and used in accordance with institutional and national regulations. No procedures on live animals were performed at our institution.

**Competing interests.** The authors declare no competing interests.

## Methods

**Methodology**

**Construction of a Dual-Expression Vector**

A synthetic cassette was designed and synthesised by Genscript, consisting of a T7 promoter followed by the *Xenopus* β-globin 5′ UTR, a first XcmI site, a reverse-oriented LacZ cassette containing a multiple cloning site (MCS) with several restriction enzyme sites, a second XcmI site to enable TA cloning, and the *Xenopus* β-globin 3′ UTR. This cassette was initially cloned into the pUC57 vector. The insert was then excised using SacI and AgeI and subcloned into the pcDNA3.1(+) backbone, which provides a CMV promoter upstream of the MCS and a bovine growth hormone (bGH) polyadenylation signal downstream. Colonies with successfully cloned LacZ fragments produced blue colonies on LB agar plates supplemented with ampicillin and 40 μg/mL X-gal. Following bacterial culture and plasmid miniprep, the construct (pXeDNA3.1-LacZ) was verified by whole-plasmid sequencing (Eurofins). The complete plasmid sequence is provided as an Extended Data file in FASTA format, and annotated maps were generated using SnapGene software (GSL Biotech, version 8.1.1).

**Cloning of Human TRPV1 Gene into pXeDNA3.1-LacZ**

The HsTRPV1 gene was amplified from cDNA derived from U373-MG cells. The primers used for amplification were: forward primer (5′-ACCATGAAGAAATGGAGCAGC-3′) and reverse primer (5′-TCACTTCTCCCCGGAAGCG-3′). PCR was performed using VeriFi™ Red PCR Master Mix (PCRBIO, Cat. No. PB10.41). Following amplification, 1 μL of Taq DNA polymerase (NEB, Cat. No. M0273) was added directly to the PCR reaction mixture and incubated at 72 °C for 15 minutes to add adenine (A) overhangs required for subsequent TA cloning. The pXeDNA3.1-LacZ vector was linearised using *XcmI* (NEB, Cat. No. R0533), generating complementary thymine (T) overhangs. Digestion was confirmed by agarose gel electrophoresis. The linearised vector was purified using the Monarch® DNA Gel Extraction Kit (NEB, Cat. No. T1020), following the manufacturer’s protocol. The PCR-amplified HsTRPV1 insert was then ligated into the linearised pXeDNA3.1-LacZ vector using T4 DNA ligase (NEB, Cat. No. M0202). The ligation mixture was transformed into competent *E. coli* cells and plated onto LB agar plates supplemented with ampicillin and X-gal for blue/white screening. We named this vector “pXeDNA3.1-HsTRPV1”.

**Bioluminescence Assay in HEK293G5A cells**

The full protocol for receptor deorphanisation in HEK cells has been described previously and scaled for high-throughput GPCR screening (Thiel, Yañez-Guerra and Jékely, 2023; Thiel et al., 2024, Yanez-Guerra et al., 2025). Here, we adapt that workflow for ion-channel assays and summarise the protocol used in this study below. HEK293 cells stably expressing the chimeric GFP–Aequorin protein G5A (HEK293G5A) were cultured in DMEM supplemented with 4.5 g/L glucose, L-glutamine, sodium pyruvate, and 10% FBS under 5% CO₂ at 37 °C (Gibco. Cat No. 11995073). Cells were seeded into white, clear-bottom 96-well plates and grown for two days. At ~90% confluency, transfection was performed using polyethyleneimine 1mg/ml (PEI, MW 40,000; Polysciences, Cat. No. 24765). For transfection, the culture medium was exchanged for 90 µL of Advanced Medium supplemented with 5% FBS. A DNA–PEI complex was prepared by mixing 60 ng of the pXeDNA3.1-HsTRPV1 plasmid DNA with 0.35 µL PEI in 10 µL Opti-MEM (without serum or phenol red) per well, incubated for 10 minutes at room temperature, and then added to the wells. Cells were returned to the incubator and allowed to express for 48 h. Two days post-transfection, the medium was replaced with 50 µL per well of 1x HBSS (140 mM NaCl, 5 mM KCl, 1 mM CaCl₂, 0.4 mM MgSO₄, 0.5 mM MgCl₂, 0.3 mM Na₂HPO₄, 0.4 mM KH₂PO₄, 6 mM glucose, 4 mM NaHCO₃) without phenol and without FBS, supplemented with 4 µM coelenterazine H (Promega Cat. No. S2011). Cells were incubated in the dark at 37 °C for 2 h to allow aequorin loading. During this incubation, a separate reagent plate was prepared containing capsaicin serially diluted in 1× HBSS across the concentration range 10⁻⁴ to 10⁻¹¹ M. Following the incubation period, bioluminescence measurements were performed on a FlexStation 3 Multi-Mode Microplate Reader, which automatically dispensed the prepared capsaicin dilutions into the wells containing aequorin-loaded cells. Luminescence signals, corresponding to Ca²⁺ influx, were recorded in real time. Raw luminescence data were converted into tibble format and analysed in R using the drc package to fit dose–response curves and estimate EC₅₀ values. Data were generated from three independent transfections (biological replicates), each performed on separate plates, and for each concentration three wells were assayed (technical replicates). Thus, each concentration point reflects the mean of nine measurements. Data processing followed the publicly available R script published before (Yañez Guerra and Zandawala, 2023) , which can be accessed via GitHub at https://github.com/Imnotabioinformatician/Branchiostoma_Dose_response_curves

**Electrophysiology of *Xenopus* Oocytes:**

Defolliculated *Xenopus laevis* oocytes were obtained from EcoCyte Bioscience and maintained in ND96 solution as follows (in mM): 96 NaCl, 1 MgCl _2_ , 5 HEPES, 1.8 CaCl _2_ , and 2 KCl. The pXeDNA3.1-HsTRPV1 plasmid was linearised using *PmeI* (Thermo Fisher Scientific Cat. No. ER1341), followed by DNA purification (Zymo Cat. No. D4013) and elution in RNAase free water. cRNA was synthesised using the T7 mMessage mMachine transcription kit (Thermo Fisher Scientific) according to the manufacturer's protocol (with overnight incubation at 37 °C). RNA was purified using the GeneJET RNA purification kit (Thermo Fisher Scientific) and quantified using a NanoDrop spectrophotometer. Oocytes were injected with 50 nl of 500 ng/µl RNA using the Roboinject system (Multi Channel Systems). Injected oocytes were incubated at 18 °C in ND96 solution until the day of recording, either 1 or 2 days postinjection.

Two-electrode voltage-clamp (TEVC) recordings were conducted using the Roboocyte2 System (Multi Channel Systems). Measuring head electrode resistance was approximately 400-1200 kΩ, pulled on a P-97 Micropipette Puller (Sutter Instrument). Electrodes contained AgCl wires backfilled with a 1 M KCl and 1.5 M KAc mixture. Oocytes were clamped at -60 mV during continuous recording at 500 Hz. 100 mM stock of capsaicin was made in ethanol. Capsaicin application lasted for 7 s, followed by a 60 s wash with ND96, perfusion speed was set to approximately 7 ml/min throughout. Two batches of oocytes were used: one batch for recordings after 1 d incubation and another batch for recordings after 2 d incubation. Uninjected oocytes were also tested after 1 d incubation and did not respond to capsaicin. Data was analysed using the Roboocyte2+ software and plotted in GraphPad Prism.

## Reagents

**Table:** 

**STRAIN/CELL LINE** &nbsp;	**DESCRIPTION** &nbsp;	**AVAILABLE FROM** &nbsp;	**CAT. NO.** &nbsp;
*Xenopus* laevis oocytes&nbsp;	Stage V-VI ooctyes for TEVC recordings&nbsp;	EcoCyte Bioscience&nbsp;	&nbsp;
HEK293G5A cells&nbsp;	Human embryonic kidney cells stably expressing GFP-Aequorin chimera&nbsp;	Angio-proteomie&nbsp;	cAP-0200AEQ-Cyto&nbsp;
U373-MG cells&nbsp;	Human glioblastoma astrocytoma cell line (source of HsTRPV1 cDNA)&nbsp;	ATCC&nbsp;	HTB-17&nbsp;
**REAGENT/ENZYME** &nbsp;	**DESCRIPTION** &nbsp;	**AVAILABLE FROM** &nbsp;	**CAT NO.** &nbsp;
XcmI&nbsp;	Restriction enzyme used for linearization&nbsp;	NEB&nbsp;	R0533&nbsp;
SacI&nbsp;	Restriction enzymes used for cloning&nbsp;	NEB&nbsp;	R0156&nbsp;
AgeI&nbsp;	Restriction enzymes used for cloning&nbsp;	NEB&nbsp;	R3552&nbsp;
PmeI&nbsp;	Restriction enzyme used for plasmid linearisation&nbsp;	Thermo Fisher Scientific&nbsp;	ER1341&nbsp;
T4 DNA ligase&nbsp;	Ligation of HsTRPV1 insert&nbsp;	NEB&nbsp;	M0273&nbsp;
Taq DNA polymerase&nbsp;	Used to add A-overhangs for TA cloning&nbsp;	NEB&nbsp;	M0202&nbsp;
VeriFi™ Red PCR Master Mix&nbsp;	High-fidelity PCR mix&nbsp;	PCRBIO&nbsp;	PB10.41&nbsp;
Monarch® DNA Gel Extraction Kit&nbsp;	DNA purification&nbsp;	NEB&nbsp;	T1020&nbsp;
DNA Clean & Concentrator-5&nbsp;	DNA clean up&nbsp;	Zymo&nbsp;	D4013&nbsp;
mMESSAGE mMACHINE T7 Kit&nbsp;	cRNA synthesis&nbsp;	Thermo Fisher Scientific&nbsp;	AM1344&nbsp;
GeneJET RNA Purification Kit&nbsp;	RNA purification&nbsp;	Thermo Fisher Scientific&nbsp;	K0791&nbsp;
**REAGENT/CHEMICAL** &nbsp;	**DESCRIPTION** &nbsp;	**AVAILABLE FROM** &nbsp;	**CAT NO.** &nbsp;
Polyethylenimine (PEI)&nbsp;	Transfection reagent&nbsp;	Polysciences&nbsp;	24765&nbsp;
Coelenterazine.H&nbsp;	Aequorin substrate for bioluminescence assay&nbsp;	Promega&nbsp;	S2011&nbsp;
Capsaicin&nbsp;	HsTRPV1 agonist&nbsp;	Sigma-Aldrich&nbsp;	M2028&nbsp;
X-gal&nbsp;	Substrate for blue/white screening&nbsp;	Sigma-Aldrich&nbsp;	B4252&nbsp;
Amipicilin&nbsp;	Selection antibiotic&nbsp;	Sigma-Aldrich&nbsp;	A9518&nbsp;
**PLASMID** &nbsp;	**CONSTRUCT** &nbsp;	**AVAILABLE FROM** &nbsp;	**ID** &nbsp;
pXeDNA3.1-LacZ&nbsp;	CMV promoter, *Xenopus* beta-globin UTRs, LacZ XcmI sites&nbsp;	Addgene&nbsp;&nbsp;	242746&nbsp;
pXeDNA3.1-HsTRPV1&nbsp;	Human TRPV1 cloned into pXeDNA3.1-LacZ&nbsp;	Addgene&nbsp;&nbsp;	244454&nbsp;
